# Early infant diagnosis: gateway to survival for HIV positive infants

**DOI:** 10.1186/1471-2334-14-S3-E13

**Published:** 2014-05-27

**Authors:** Jayanthi Shastri, Sachee Agrawal, Sandhya Sawant, Manish Pathak

**Affiliations:** 1 Department of Microbiology, T.N.Medical College and B.Y.L.Nair Charitable Hospital, Mumbai, India

## Background

Under PPTCT, babies born to sero-positive mothers were followed up and tested at 18 months of age. Under Early Infant Diagnosis (EID) program, HIV exposed babies are subjected to HIV-1 DNA PCR on DBS and whole blood as early as 6 weeks of age followed by initiation of ART.

## Methods

Infants born to HIV positive mothers were tested according to NACO guidelines. Infants <6 months of age (n=136) were tested by DBS HIV-1 DNA PCR; DBS positive were confirmed by whole blood PCR. Infants 6 to 18 months (n=68) were tested by antibody test and if positive were confirmed by DBS HIV-1 DNA PCR. Detailed history including type of delivery, Single Dose Nevirapine (SDN) and breast feeding was taken.

## Results

The HIV transmission rate was 11.28% (23/204); 13 infants <6 months of age and 10 infants ≥6 months of age. In infants <6 months of age, who did not receive SDN the positivity was 38.46% (5/13) whereas in those who received SDN it was 6.5% (8/123), [*p*=0.0012]. In infants ≥6 months the positivity rate was significantly higher in breast fed 60% (6/10) as compared to non breast fed 6.9% (4/58),[*p*=0.0001]. 60.9% (14/23) infants were delivered at home and did not receive SDN.

## Conclusion

In resource limited settings, SDN given to mother in labor is a good option.. Mothers intending to continue breast feeding should be provided extended ART. EID and prompt ART institution ensures favourable outcomes in HIV exposed infants.

